# Detecting Attomolar Concentrations of Interleukin IL-17A via Pollen-Based Nanoplasmonic Biochips

**DOI:** 10.3390/bios15030161

**Published:** 2025-03-03

**Authors:** Chiara Marzano, Rosalba Pitruzzella, Francesco Arcadio, Federica Passeggio, Mimimorena Seggio, Luigi Zeni, Laura Pasquardini, Nunzio Cennamo

**Affiliations:** 1 Department of Engineering, University of Campania Luigi Vanvitelli, Via Roma 29, 81031 Aversa, Italy; chiara.marzano@unicampania.it (C.M.); rosalba.pitruzzella@unicampania.it (R.P.); francesco.arcadio@unicampania.it (F.A.); federica.passeggio@unicampania.it (F.P.); mimiseggio@gmail.com (M.S.); luigi.zeni@unicampania.it (L.Z.); 2 Indivenire Srl, Via Sommarive 18, 38123 Trento, Italy

**Keywords:** nanoplasmonics, natural periodical nanostructures, pollen, biosensors, interleukin IL-17A

## Abstract

Interleukins are involved in several diseases and cancers, and their detection and monitoring are of great interest. Their low abundance and short half-lives suggest the need to develop rapid, specific, and highly sensitive detection platforms, easily integrable in point-of-care (POC) systems. Among the other interleukins, interleukin IL-17A is associated with inflammations, neurodegenerative diseases, and cancers, and no biosensors have been previously reported for its detection. In this work, for the detection of IL-17A, a highly sensitive nanoplasmonic sensor based on natural nanostructures like pollen shells, covered by a gold film and a bio-receptor layer, is presented. Hybrid plasmonic modes are exploited to reach high sensitivity without using costly techniques to fabricate periodic nanostructures, such as electron beam lithography. A transparent amino-modified glass substrate is functionalized with carboxylic activated pollen via carbodiimide chemistry. Then, the pollen-based nanostructures are covered by a gold film and derivatized by an immuno-layer specific to IL-17A recognition. The developed IL-17A biosensor is monitored via a simple, small-sized, and low-cost experimental setup, demonstrating high selectivity, a fast response time of about five minutes, and sensitivity with a limit of detection in the ag/mL concentration range. The biosensor allows for the detection of IL-17A in complex solutions thanks to the possibility of high dilution, an advantageous aspect to POC systems.

## 1. Introduction

Cytokines are low-molecular-weight proteins that play a crucial role in several inflammatory events and cancer, promoting disease proliferation; at the same time, the inhibition of specific cytokines can be an effective therapeutic treatment. Moreover, the cytokine storm has also been observed as a response to COVID-19 infection [1]. Among the other cytokines, the interleukin family is of growing interest in the medical domain; the widely studied interleukins are IL-6, IL-8, IL-1, etc., but IL-17 has received increased interest over the years.

Interleukin (IL)-17 was originally isolated in 1993 from a rat–mouse T-cell hybridoma and recognized as a cytokine two years later [2,3]. Today, six different homologous molecules are known, and the most important are IL-17A and IL-17F [2,4]. IL-17A is involved in several diseases [3], like inflammatory diseases [5], human rheumatoid arthritis, psoriasis [6], neurodegenerative diseases [7], and different kinds of cancers [8,9]. Moreover, IL-17A has been found to promote neuronal and tissue regeneration and host defense, showing that positive effects can also be attributed to this interleukin [10,11]. The inhibition of this interleukin, via a specific receptor or antibody, is an established method to treat the diseases [3,4,6,12].

The detection of interleukins, in general, is a highly necessary and extremely critical task due to their low abundance (in fM or pM ranges) in body fluids, the short half-life of some of these molecules, and the dynamic secretion that can also be caused by the procedures for sample collection, inducing false positive results [13].

With respect to standard assays like the enzyme-linked immunosorbent assay (ELISA) test that requires a lot of time and reagents, biosensors are characterized by a fast response, a high sensitivity, an easy operation. They are easily integrable in point-of-care (POC) devices, which can be used to monitor the progress of the disease or the evolution of the selected treatment directly at the bed site. Among the other kinds of biosensors [13,14,15], plasmonic-based biosensors are widely explored as sensitive devices to achieve a low limit of detection (LoD) through label-free measurement and real-time monitoring [16]. Focusing on interleukins, several detection platforms have been developed with high performances [17,18,19,20,21,22,23,24,25,26,27,28,29].

The low abundance of interleukins is one of the main issues that need to be solved in their detection. Normal levels in healthy people are in the pg/mL range and rise to the ng/mL range when diseases are present in blood or serum samples; however, these values can go down to fg/mL in other biological fluids like saliva, sweat, or urine [15]. A possible solution is to use amplification systems [30], but the possibility of reaching the required LoD with a reagentless system, characterized by a fast response and a high specificity, remains the main challenge.

In this work, a pollen-based nanoplasmonic probe, recently developed by our group [31], is applied for the first time to the detection of IL-17A interleukin via a specific bio-receptor layer. The pollen-based plasmonic probe can be used to excite hybrid plasmonic phenomena originated by the pseudo-periodic nanostructures of the pollen tips [31]. These plasmonic phenomena produce an ultra-high sensitivity, which is useful for detecting targets at an attomolar level.

Most of the literature based on plasmonic devices is related to the detection of IL-6 interleukin, while only two biosensors related to IL-17A are reported and are designed to recognize not the protein itself but its receptor [32,33]. No example of the IL-17A protein itself has been reported up to now.

This work presents several experimental tests in order to demonstrate the direct detection of IL-17A interleukin through a high-sensitivity nanoplasmonic platform based on pollen’s natural nanostructures, reaching a LoD never achieved before in interleukin detection. It uses a plasmonic-based platform, without amplification, and results are available in a few minutes. There is also the possibility to use the platform on a buffer solution, since it is possible to dilute the complex samples without losing performance.

## 2. Materials and Methods

### 2.1. Materials

Defatted sunflower pollen grains are purchased from Greer Labs (Lenoir, NC, USA). 1-ethyl-3-(3-dimethylaminopropyl) carbodiimide hydrochloride (EDC, 22980) and N-hydroxysulfosuccinimide (Sulfo-NHS, 24510) are purchased from Fisher Scientific Italia (Milan, Italy). α-lipoic acid, ethanolamine, Tween-20, potassium hydrochloride beads, and powder for the buffer solution are purchased from Sigma Aldrich (Milan, Italy). Recombinant human IL-17A protein (ab282392), recombinant human IL-1 beta protein (ab259387), recombinant human IL-18 protein (ab316093), and rabbit polyclonal anti-IL-17A antibody (ab79056) are purchased from Abcam (Cambridge, UK).

Aminosilane-coated glass slides Nexterion A+ are purchased from Schott Technical Glass Solutions GmbH (Jena, Germany).

### 2.2. Plasmonic Pollen-Based Chip Preparation

**Figure 1:**
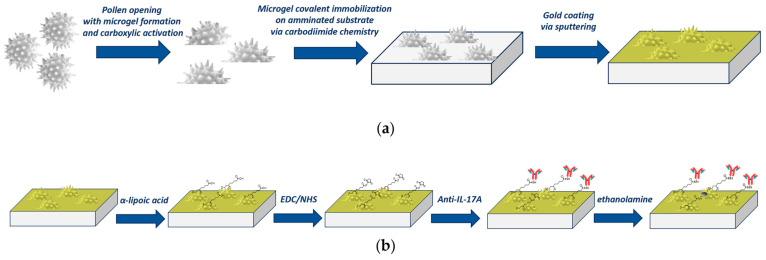
(**a**) Outline of the production steps to achieve pollen-based nanoplasmonic platforms. (**b**) Outline of the functionalization steps of the pollen-based nanoplasmonic chip.

The chip preparation starts from the basic treatment of defatted pollen to obtain carboxylic-activated opened pollen structures using a known method reported in the literature [34,35] and subsequently used by our group [31], as summarized in the outline shown in Figure 1a. Briefly, 1 g of defatted pollen is weighted in a round-bottom flask and filled with 10 mL of 10% (*w*/*v*) KOH aqueous solution, and left to reflux for 2 h at 80 °C under magnetic stirring. Then, a centrifugation (ALC PK 120 R) at 3500 rpm for 5 min is applied, and after being washed four times in 20 mL of 10% KOH, the pellet is resuspended in 10 mL of fresh 10% KOH solution and incubated statically at 80 °C for 6 h. Finally, the solution is centrifuged in 40 mL of MilliQ water four times until the pH value reaches neutrality. The carboxylic-activated pollen is then immobilized on an aminotreated glass substrate via carbodimide chemistry. Briefly, 200 mg of pollen is incubated in 40 mM of EDC and 10 mM of sulphoNHS in MES buffer (50 mM MES, pH 5.5) for 30 min under agitation. The solution is then centrifugated at 3500 rpm (ALC PK 120 R) for 5 min, and the pellet is resuspended in phosphate buffer (PBS) and incubated on an aminosilane-treated glass substrate for 1 h. The PBS is made of 10 mM phosphate buffer, 138 mM NaCl, and 2.7 mM KCl, and has a pH of 7.4. After washing and drying, the substrate with covalently immobilized pollen is covered by 45 nm of gold using a sputter coater (K-575X, Quorumtech, Lewes, UK) [31].

### 2.3. Pollen Density Estimation

The high autofluorescence of the pollen is used to estimate its density on the amino-functionalized surfaces. A Leica DMLA fluorescence microscope equipped with a mercury lamp and a fluorescence filter L5 (Leica Microsystems, Wetzlar, Germany) is used, measuring the signal with a 10× magnification objective through a cooled CCD camera (DFC420C, Leica Microsystems, Wetzlar, Germany) and analyzing images with the Fiji software (Java version 8) [36].

### 2.4. Surface Functionalization of the Pollen-Based Nanoplasmonic Chip

The pollen-based plasmonic chips, obtained as reported in Figure 1a, are sequentially cleaned with filtered Milli-Q water (3 washes, 5 min each time) and 8% ethanol/Milli-Q water (3 times for 5 min) and then treated for 18 h at room temperature with lipoic acid (0.3 mM in 8% ethanol solution) to obtain a surface exposing carboxyl groups. The activation is performed via carboimide chemistry (NHS/EDC, 200 mM/50 mM in PBS pH 7.4) for 20 min at room temperature. After three washing steps in PBS, incubation with 10 μL of anti-IL-17A antibody (0.5 mg/mL) for 2 h at room temperature is performed. To remove the non-covalent bond between the antibody and the surface, three washing steps (3 × 5 min, in PBS + 0.005% Tween 20) are performed, and a final passivation for 30 min at room temperature with 1 M ethanolamine, pH 8.0, is applied. Figure 1b summarizes the steps required for the functionalization process.

### 2.5. Optical Setup

**Figure 2:**
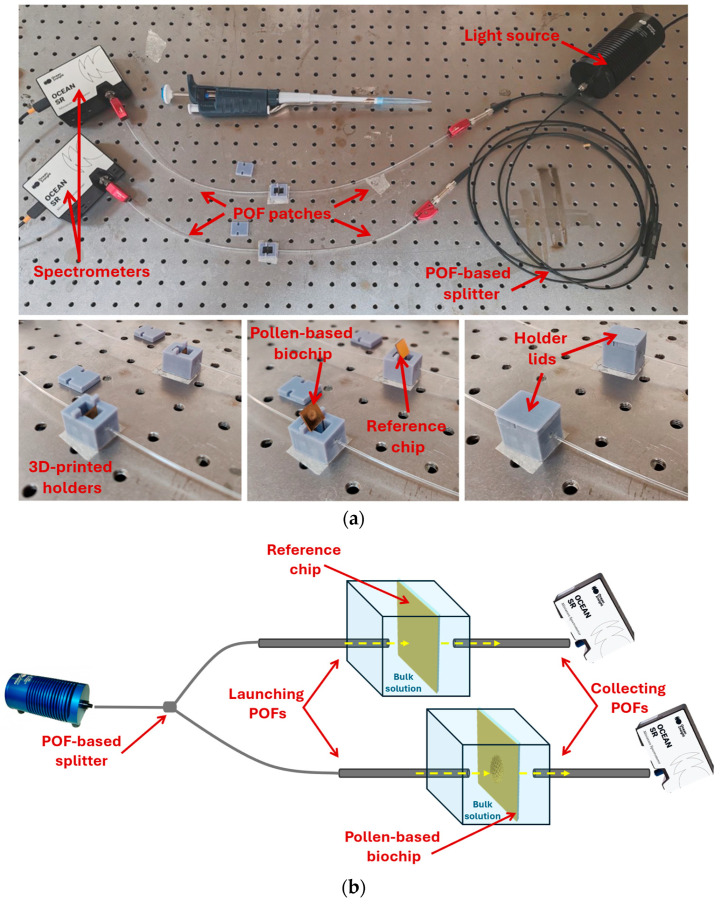
(**a**) Picture of the complete experimental setup used to test the pollen-based nanoplasmonic sensors (above) and enlargement of the two holders used (bottom). (**b**) Outline of the optical paths (yellow dash lines) in both reference and pollen-based chips.

Figure 2a displays a picture of the optical setup used for the measurements. Using an optical splitter (50:50) based on Plastic Optical Fibers (POFs), a broadband white light source (HL2000-LL, Ocean Insight, Orlando, FL, USA) is coupled in input to the measuring cells (3D-printed holders) via two POF patches (PMMA core with a total diameter of 1 mm). Through the POFs, the light is guided into the reference chip (a similar chip without the nanostructures) and into the pollen-based biochip [31]. The two chips are inserted in custom-designed 3D-printed holders (the grey box in Figure 2a) with the testing solution. Both chips are positioned in an orthogonal way with respect to the direction of the input/output light. The normal incidence of the light on the reference chip does not excite any plasmonic effect, and its signal is used to normalize the spectra from the pollen-based biochip. On the other side, in the output of the 3D-printed holders, two similar POF patches are used to collect and send the transmitted light from both chips to two similar spectrometers (SR-6VN500, purchased by Ocean Optics, Orlando, FL, USA), as shown in Figure 2a [31,37]. The optical paths for the reference and pollen-based chips are shown in the outline of Figure 2b.

In this work, with respect to the previous nanoplasmonic sensor configurations [31,37], two similar novel 3D-printed holders have been developed and used instead of one bigger holder to reduce the liquid volume under test. In fact, the solutions at different concentrations are dropped only in the measuring cell with the pollen-based biochip.

### 2.6. Measurement Protocol and Data Analysis

The measuring protocol consists of filling the holder of the reference chip with 900 μL of the bulk solution (phosphate buffer), whereas the other one contains 900 μL of solution with the analyte (at different concentrations), and leaving them for 5 min as incubation time. After the incubation time and washing with PBS three times, the transmitted spectra are acquired using PBS as the bulk solution. Therefore, the proposed biosensor can perform the analysis in a short time—less than 10 min—allowing the monitoring of molecules characterized by a short half-life and the following of the dynamic secretion of these molecules (such as interleukins). The spectra are obtained by normalizing the transmitted spectra recorded at different interleukin concentrations from 0.1 aM to 1000 aM. The dose–response curve is modelled using the Langmuir fitting (Equation (1)):

(1) $$\left|{∆\lambda }_{c}\right|=\left|\lambda_{c}-\lambda_{0}\right|=\left|{∆\lambda }_{max}\right|\cdot \frac{c}{K+c}$$

where c refers to the interleukin concentration, *λ*_c_ represents the plasmonic resonance wavelength at concentration c, *λ*_0_ is the resonance wavelength of the blank, and Δ*λ*_max_ indicates the difference between the resonance wavelength at saturation value and the blank. The Langmuir model allows us to determine the affinity constant of the bioreceptor (since K = 1/K_aff_, where K_aff_ represents the affinity constant). At a very low analyte concentration, i.e., c ≪ K, Equation (1) can be considered linear, and the slope of this linear function (|Δλ_max_|⁄K) is defined as sensitivity at low concentrations (S*_low c_*). In order to estimate the LoD, it is necessary to calculate the ratio of three times the standard deviation of blank (reported in Langmuir fitting parameters) and S*_low c_*, as defined in Equation (2):

(2) $$LoD=\frac{3\times St.\;dev.\;(|{\Delta \lambda }_{0}|)}{S_{low\;c}}$$

## 3. Results and Discussion

In the following paragraphs, the main findings of the pollen-based biosensor are reported, starting from a characterization of the surface through fluorescence, in order to estimate the pollen density on the biochip, and then presenting the biosensor performances in terms of sensitivity and specificity. This is the first biosensor specific to IL-17A, therefore the performances of our biosensor are compared with plasmonic-based biosensors specific to other interleukins.

### 3.1. Pollen Density Measurements

**Figure 3:**
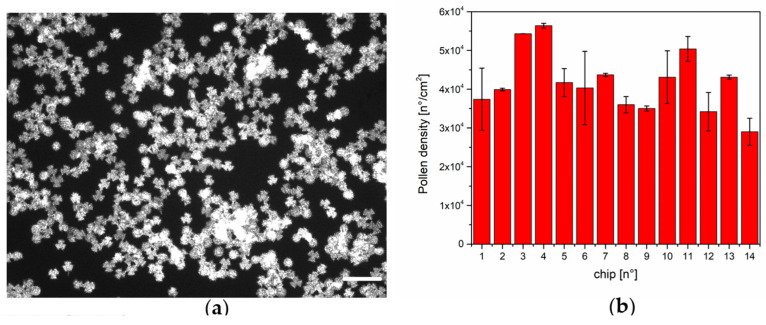
Pollen density: (**a**) Autofluorescence of the pollen on amino-coated substrate, scale bar is 100 µm; (**b**) Pollen density estimation on 14 different amino-coated substrates.

The natural autofluorescence of the pollen was used to characterize the pollen density on the amino-coated glass substrate. Figure 3 shows an example of the pollen distribution on the substrate (Figure 3a) and the numerical evaluation of the pollen density on 14 different substrates (Figure 3b). The procedure is reproducible in different experimental sessions (as reported in [31]), with a mean density in the range of 4 × 10^4^ pollen/cm^2^.

### 3.2. IL-17A Detection via Pollen-Based Biochips

**Figure 4:**
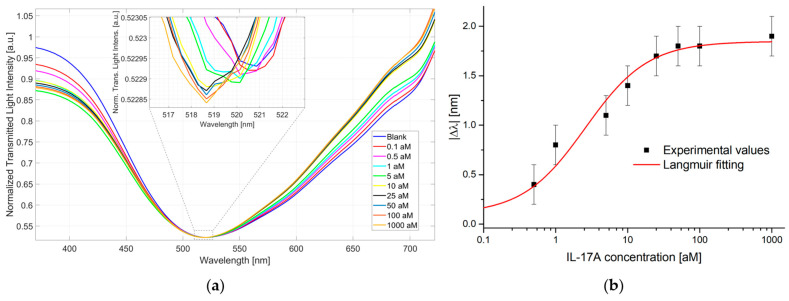
Interleukin 17A detection in the buffer: (**a**) Plasmonic spectra obtained via different IL-17A concentrations in the buffer from 0.1 aM to 1000 aM, and inset shows an enlargement of the resonance peak; (**b**) Dose–response curve: wavelength shift with respect to the blank (absolute module) as a function of IL-17A concentration in a semilog scale, together with the Langmuir fitting of the experimental values and error bars (maximum measured standard deviation).

The pollen-based biosensor was tested in the concentration range from 0.1 to 1000 aM for the detection of IL-17A in PBS. In particular, three similar pollen-based biochips were developed and tested in the same conditions to demonstrate the reproducible abilities of the sensor biochip, sensor system, and sensing approach. The pollen-based biosensor is able to detect ultra-low concentrations (at the attomolar level) of the analyte, monitoring the resonance wavelength blue shift with a maximum extension of about 2 nm, as reported in Figure 4a. In particular, Figure 4a reports the plasmonic spectra obtained at different IL-17A concentrations (from 0.1 aM to 1000 aM), highlighting in the inset the resonance peak evolution, whereas Figure 4b shows the dose–response curve obtained via the absolute wavelength shift with respect to the blank (solution without the analyte) as a function of the analyte concentrations in a semilog scale. The standard deviations of the sensor system are related to three similar measurements carried out in the same conditions via three similar biochips. More specifically, the error bar in Figure 4b is the maximum measured standard deviation equal to 0.2 nm. The experimental values were fitted to the Langmuir model (Equation (1)) using OriginPro software (version 9.2, Origin Lab. Corp., Northampton, MA, USA) (Figure 4b). The parameters obtained for the Langmuir fitting of the IL-17A dose—response curve shown in Figure 4b are reported in Table 1.

Based on the values reported in Table 1, as reported in Section 2.6, it is possible to estimate the S*_low c_* that resulted as being equal to 0.71 nm/aM, the K_aff_ of the bioreceptor as being equal to 0.39 [aM]^−1^, and the LoD of the biosensor, calculated using Equation (2), as 0.46 aM (6.9 ag/mL).

### 3.3. Selectivity Tests of the IL-17A Pollen-Based Plasmonic Biosensor

A key point for an efficient biosensor is its ability to recognize the target even in the presence of other molecules. Often, the analyte is contained in complex biological matrices like serum, plasma, entire blood, saliva, sweat, or urine. The IL-17A pollen-based biosensor was tested against possible interferents, like interleukin IL-1β and IL-18.

**Figure 5:**
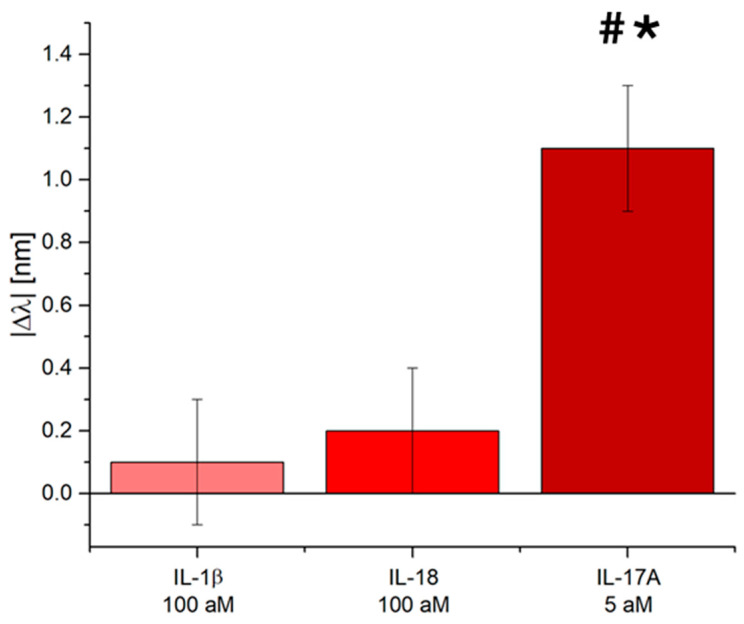
Selectivity test: pollen-based nanoplasmonic biosensor response to IL-1β and IL-18 at 100 aM and to IL-17A at 5 aM. One-way ANOVA # *p* < 0.05 vs. IL-1β and * *p* < 0.05 vs. IL-18.

Figure 5 shows the absolute value of the resonance wavelength shift with respect to the blank due to the sensitive sensor region interaction with the other two interleukins (IL-1β and IL-18), both at a concentration equal to 100 aM. As shown in Figure 5, other interleukins did not produce any significant plasmonic resonance wavelength shift if compared to the one obtained by the IL-17A tested at a much lower concentration—20 times lower, equal to 5 aM. All the tests were performed in a buffer solution because the obtained biosensor sensitivity allows for the detection of the IL-17A in different complex matrices diluted to several orders of magnitude, resulting in a solution like a buffer solution.

This confirmed not only the bioreceptor’s high affinity but also the functionalization process’s goodness, which avoids nonspecific adsorption on the nanoplasmonic chip.

### 3.4. Discussion: Comparison with the State-of-the-Art

Some biosensors related to the detection of IL-17A are reported in the literature, and all are related to its receptor IL-17RA. An aptasensor against IL-17RA, the most active receptor through which IL-17A expresses its function, is reported by Jo and co-workers [32]: through impedimetric measurements, the authors achieved a LoD of 2.13 pg/mL, lower than commercial ELISA assays. Smejkal and co-workers worked on the development of small recombinant protein binders as an alternative to antibodies, tested on IL-23R and IL-17RA with high affinity [33]. No works have been found on the IL-17A interleukin. Therefore, we generally refer to interleukin detection to compare the proposed biosensor with the state-of-the-art one.

Table 2 reports the performances of several plasmonic platforms in the detection of interleukins.

Most of the reported works are based on localized surface plasmon resonance (LSPR) and surface plasmon resonance (SPR). LSPR probes allow for an enhancement of the plasmonic effect through the use of nanostructures; the most widely used probes are based on gold nanoparticles [18,20,26,38], but examples using nanodimers [39], nanorods [17,23,25], nanopillar [27] or nanograting [22] are also reported. With respect to the LSPR and SPR plasmonic systems, the pollen-based plasmonic platform is based on pseudo-periodic nanostructures that can be used to excite hybrid plasmonic phenomena: when surface plasmons (SPs) and localized SPs (LSPs) are simultaneously excited by the nanostructures, their joint interaction can trigger plasmonic hybrid modes [31,37].

Other platforms take advantage of the plasmonic effect induced by a metallic layer to enhance the Raman signal in Surface Enhanced Raman Scattering (SERS) spectroscopy [21,28,29,39] or are based on photonic crystals [23]. However, most proposed devices require complex and not easily integrable detection systems like microscopy [18,26,38] or are based on amplification systems like secondary antibodies conjugated to gold nanoparticles [20,29].

With respect to these plasmonic biosensor systems (as summarized in Table 2), an innovative biosensor that is potentially low-cost and easily integrable in a POC device for single-molecule detection is proposed in this work.

## 4. Conclusions

The detection of interleukins is in high demand since their secretion in body fluids is related to several different diseases and consequences of medical treatments. Moreover, their low abundance and short half-lifse make their detection critical and require the development of highly sensitive and fast devices integrable in a POC device. In this context, biosensors can offer a successful answer. Here, for the first time, a biosensor for the detection of IL-17A at a single-molecule level (at a zepto-atto molar level) is reported using a highly sensitive plasmonic platform based on hybrid modes produced by pollen nanostructures. The proposed biosensor presents an ultra-low detection limit that has never been achieved before for interleukin detection using plasmonic principles without amplification systems in a short time (less than 10 min). The biosensor also demonstrates a high specificity towards its analyte with an LoD in the ag/mL range, suggesting that it is possible to analyze saliva or sweat where the analyte is present in very low concentration (fg/mL), diluting the sample several orders of magnitude in buffer and cancelling the real matrix effect.

## Figures and Tables

**Table 1:** Langmuir fitting parameters determined using Equation (1) on experimental values (see Figure 4b).

|Δλ_0_| [nm]	|Δλ_max_| [nm]	K [aM]	R^2^
0.10 ± 0.11	1.85 ± 0.07	2.59 ± 0.77	0.97

**Table 2:** Comparison of different plasmonic-based biosensors for interleukin detection.

Biosensor Principle	Interleukin	LoD	Reference
LSPR of single gold nanoparticles	IL-6	-	[38]
Optical waveguide with plasmonic surfaces based on gold nanodimers	IL-6	-	[39]
LSPR microfluidic platform coupled with gold nanorods	IL-2, IL-4, IL-6, IL-10	20.56 pg/mL, 4.6 pg/mL, 11.29 pg/mL, 10.97 pg/mL	[25]
LSPR immunoassay with silver-enhanced gold nanoparticles conjugated to secondary antibodies	IL-6	50 pg/mL	[26]
Plasmonic droplet device	IL-8	7.2 ng/mL	[17]
Nanoimprinted gold-capped nanopillar structures on polymer for LSPR detection	IL-6	10 ng/mL	[27]
SERS based immunoassay	IL-8	6.2 pg/mL	[28]
Paper-based SERS assay	IL-10	0.1 pg/mL	[29]
Dark field microscopy coupled with LSPR effect of Au nanoparticles	IL-6	7 pg/mL	[18]
Plasmonic nanocrystals based in Blu-ray optical disc	IL-6	0.03 ng/mL	[19]
Plasmonic effect of Au nanoparticles on paper-based device	IL-6	0.1 pg/mL	[20]
Microfluidic SERS biosensor based on magnetic/plasmonic nanostirrer embedded with Raman reporter	IL-6	0.8 pg/mL	[21]
SPR-POF platform	IL-6	0.84 ng/mL	[22]
gold nanograting GNG		14.28 fg/mL	
Plasmonic gold nanorods coupled with photonic crystal	IL-6	10 fg/mL	[23]
Titanium oxynitride nanofilm	IL-6	6.3 fg/mL	[24]
Pollen-based biosensor	IL-17A	6.9 ag/mL	Present study

## Data Availability

The data are available upon reasonable request from the corresponding author.
